# Integrated Strategy for Informative Profiling and Accurate Quantification of Key-Volatiles in Dried Fruits and Nuts: An Industrial Quality Control Perspective

**DOI:** 10.3390/foods11193111

**Published:** 2022-10-06

**Authors:** Andrea Caratti, Simone Squara, Federico Stilo, Sonia Battaglino, Erica Liberto, Irene Cincera, Giuseppe Genova, Nicola Spigolon, Carlo Bicchi, Chiara Cordero

**Affiliations:** 1 Dipartimento di Scienza e Tecnologia del Farmaco, Università degli Studi di Torino, 10125 Torino, Italy; 2 Soremartec Italia SRL, 12051 Alba CN, Italy

**Keywords:** volatile organic compounds, GC-MS, MHS-SPME sampling, accurate quantification of volatiles, dried fruits and nuts, aroma blueprint

## Abstract

Edible nuts and dried fruits, usually traded together in the global market, are one of the cornerstones of the Mediterranean diet representing a source of essential nutrients and bioactives. The food industry has an interest in the selection of high-quality materials for new product development while also matching consumers’ expectations in terms of sensory quality. In this study, walnuts (*Juglans regia*), almonds (*Prunus dulcis*), and dried pineapples (*Ananas comosus*) are selected as food models to develop an integrated analytical strategy for the informative volatile organic compounds (VOCs) quali- and quantitative profiling. The study deals with VOCs monitoring over time (12 months) and in the function of storage conditions (temperature and atmosphere).VOCs are targeted within those: (i) with a role in the product’s aroma blueprint (i.e., key-aromas and potent odorants); (ii) responsible for sensory degradation (i.e., rancidity); and/or (iii) formed by lipid autoxidation process. By accurate quantitative determination of volatile lipid oxidation markers (i.e., hexanal, heptanal, octanal, nonanal, decanal, (*E*)-2-heptenal, (*E*)-2-octenal, (*E*)-2-nonenal) product quality benchmarking is achieved. The combination of detailed VOCs profiling by headspace solid phase microextraction (HS-SPME) combined with gas chromatography-mass spectrometry (GC-MS) and accurate quantification of rancidity markers by multiple headspace-SPME (MHS-SPME) answers many different questions about shelf-life (i.e., aroma, storage stability, impact of temperature and storage atmosphere, rancidity level), while providing reliable and robust data for long-range studies and quality controls. The quantification associated with HS-SPME profiling is demonstrated and critically commented on to help the industrial research in a better understanding of the most suitable analytical strategies for supporting primary materials selection and new product development.

## 1. Introduction

Edible nuts and traditional dried fruit are considered two distinct groups of products, although usually traded together in the global market. They are one of the cornerstones of the Mediterranean diet and other regional diets; thanks also to their compositional stability along with shelf-life, they are increasingly used as snacks or ready-to-eat portioned food [1]. Consumption of edible nut and dried fruits results in documented beneficial effects on human health, depending on relatively high content of poly unsaturated fatty acids—PUFAs (e.g., linolenic and linoleic acids), lipophilic vitamins (vitamin E, group B), macro-elements (P, Mg and K) and microelements (Cu, Mn, Fe etc.) [2,3,4].

A “nut” is a fruit composed of a hard shell protecting an edible seed (rarely two) unattached or free within the ovary wall. However, in a general context, the word nut is also used to refer to edible oily kernels in-shell (e.g., almonds, Brazil nuts, cashews, hazelnuts, macadamias, peanuts, pecans, pine nuts, pistachios, and walnuts) or without hard-shell (e.g., pine nuts). Peanuts and soybeans, despite being legumes, fleshy edible berries, and seeds of several members of *Cucurbitaceae* family are also commonly considered as nuts [2,3,5]. On the other hand, a “dried fruit” is prepared from edible parts of fresh fruits, dried with natural (sun-drying) or artificial (dryers, freeze-thawing) methods or with a combination of both, until a low free water content (*a_w_*) is achieved.

Dried fruits may be whole, quarters, diced, sliced, chips, chunks, trips, balls, crisps, flakes, pieces or powdered. Thanks to their forms, they are easy to store and distribute; being a healthier alternative to salty or sugary snacks [2,3,6].

The dried fruits and edible nuts market is global: major areas are Pacific Asia followed by North America and Europe while top global importers include United Kingdom (UK), United States (US), Germany, Vietnam, and France [2]. The European Union is one of the largest world markets for nuts, receiving more than 40% of the global import. The European market trend is annually growing by 9% in value since 2013 [2]. Shelled almonds have the highest value of import (23%) followed by shelled cashew nuts (12%), shelled hazelnuts (12%) and shelled peanuts (8%).

Globally, the dried fruits market is expected to further expand in the coming years owing to rising consumer awareness as to their nutritional benefits [2]. In this context, reliable yet informative quality control (QC) procedures are required to support industrial strategies for quality evaluation of incoming batches and shelf-life monitoring within both industry storage plants and finished products.

Most of the available quality parameters and indices, are included in the official norms and standards [7,8,9,10] regulating the market classification and categorization of dried fruits and nuts, although just a few relate to chemical markers informative of the actual compositional quality and sensory profile. For this reason, industrial QC laboratories are implementing many additional tests, over the sensory evaluation performed by internal panels, to objectify the quality level of incoming batches and consequently design suitable strategies for storage and/or timely transformation into finished products.

An example is the quality assessment of hazelnut kernels [11,12,13,14,15] that is nowadays based on an integrated multimethod strategy [16,17,18]. Visual inspection targets damaged kernels [e.g., insect-damaged, rotten, twin, and yellowed kernels [17,19,20,21,22,23]], sensory evaluation reveals the presence of rancidity (taste and aroma) [24], categorization by morphological parameters (shape and kernel size/caliber) is used to direct transformation strategies, while moisture content (*a_w_*) below established levels, guarantees microbial and mold stability. Chemical markers for QC include the quali-quantitative profiling of fatty acids methyl esters (FAMEs), targeted to both free FAs (FFAs) and esterified species (EFAs) [13,25,26], accompanied by the accurate quantification of secondary products of FAs hydroperoxides degradation (i.e., hexanal, heptanal, octanal, and nonanal) [27]. Additional parameters include the free acidity [25,28], the peroxide value (PV), and the Oxitest AOCS method [29]. By this integrated yet reliable strategy, objective quality evaluation of hazelnut kernels is possible due to the analytical data transferability over the years and to many laboratories.

In this context, the current study aims at designing an integrated strategy for the QC of selected nuts (i.e., walnuts and almonds) and dried fruits (i.e., pineapple) to be used by the confectionery industry for finished products or ready-to-eat snack portions. In particular, the strategy targets informative volatile organic compounds (VOCs) known for their role in the product’s aroma blueprint (i.e., key-odorants and potent odorants) [30,31], sensory degradation (i.e., rancidity), and/or informative of the FAs autoxidation process. By detailed profiling [32] a large set of VOCs can be monitored and their evolution over time and under the different storage conditions observed. Moreover, by accurate quantitative determination of secondary products of FAs hydroperoxides degradation, the autoxidation status can be assessed. To prove the effectiveness of the proposed strategy, selected samples of commercial quality (i.e., almonds, walnuts, and dried pineapple) are monitored for twelve months of storage (t0, t4, t8, t12) and within different conditions (temperature 5° and 18 °C and atmosphere composition). The inaccuracy and inconsistency of many existing profiling procedures described in the literature, not suitable for large-scale QCs and data transferability, is proved and further commented upon.

## 2. Materials and Methods

### 2.1. Samples

Walnut (*Juglans regia* L. var. *Chandler*) samples of commercial grade were provided at time zero and after 4, 8 and 12 months of storage at 18 °C and 5 °C in normal atmosphere (NA) and under vacuum after removal of air with an inert gas (nitrogen) (SV).

Almond (*Prunus dulcis* (Mill) var. Aldrich) samples from California (US) were provided after a pasteurization step and were analyzed at time zero and after 4, 8 and 12 months of storage at 18 °C and 5 °C in normal atmosphere (NA) and under vacuum after removal of air with an inert gas (nitrogen) (SV).

Dried pineapple (*Ananas comosus* L.) samples from Costa Rica were analyzed at time zero and after 4, 8 and 12 months of storage at 18 °C and 5 °C in normal atmosphere (NA).

All samples were provided by Soremartec Italia SRL (Alba, Cuneo, Italy).

Samples belonging to the same commercial batch, were provided in three replicates (subsamples R1, R2, R3) and analyzed in duplicates randomly distributed over the entire analytical batch. Samples were ground in fine powder with a mechanical grinder with the aid of liquid nitrogen. Powdered samples were then stored at −80 °C until analysis. Samples detail and acronyms are listed in Table 1.

### 2.2. Chemicals

All chemicals were from Merck (Milan, Italy):

Pure standard solution of *n*-alkanes (from *n*-C7 to *n*-C30) for Linear Retention Indices (*I^T^_s_*) calibration according to van Den Dool and Kratz [33] were prepared in cyclohexane at the concentration of 100 mg/L and then diluted to a 10 mg/L before injection.

Pure reference standard solution of Internal Standards (ISs) α-thujone/β-thujone and methyl-2-octynoate were prepared in diethyl phthalate (99% purity) at the final concentration of 100 mg/L.

Pure reference standards for multiple headspace solid-phase microextraction (MHS-SPME), i.e., the quantification approach for secondary products of lipid oxidation, were: hexanal, heptanal, octanal, nonanal, decanal, (*E*)-2-hexenal, (*E*)-2-heptenal (*E*)-2-octenal, (*E*)-2-nonenal and (*E*)-2-decenal. Reference stock solutions (SS) for external calibration were prepared in diethyl phthalate by mixing suitable volumes of pure standards up to 10.00 g/L final concentration. Calibration mixtures were stored in sealed vials, without available headspace (HS) volume, at −18 °C for a maximum of 4 weeks. Calibration solutions were prepared to match the following absolute amounts: 5, 25, 50, 125, 250, 375, 500 and 750 ng.

### 2.3. SPME Devices and ISs Pre-Loading Conditions

HS-SPME was carried out using a Divinylbenzene/Carboxen/Polydimethylsiloxane (DVB/CAR/PDMS) d_f_ 50/30 μm and 1 cm long fiber of Supelco (Bellefonte, PA, USA).

Before sampling, the standard-in-fiber procedure was applied to pre-load all ISs on the SPME fiber. This was performed with 5.0 μL of α and β-thujone and methyl-2-octynoate standard solutions (100 mg/L) placed in a 20 mL glass sealed vial and submitted to HS-SPME at a specific temperature (see below) for 5 min.

### 2.4. HS-SPME Sampling Conditions: VOCs Profiling

Design of experiments (DoE) by Central Composite Design (CCD) strategy was conducted to define optimal sampling conditions (i.e., sample amount, sampling time, and temperature) for each sample/matrix.

In this regard, ranges for each variable were chosen according to existing literature data [4,34]. They were as follows: amount between 0.5 and 3.0 g not exceeding a proper phase ratio β; sampling time between 30 and 60 min compatible with the GC run time, sampling temperature between 30° and 60 °C to avoid the risk of sample degradation and artifacts formation at higher temperatures. Table 2 illustrates the resulting experiments matrix based on the CCD model.

To direct the choice for optimal VOCs sampling conditions, the targeted peaks cumulative chromatographic area (i.e., total ion current—TIC response) was considered, followed by a selection of a few compounds of higher relevance for the aroma blueprint [35] of the studied matrix. Further parameters used to evaluate the quality of the obtained model were:

- Average % relative standard deviation (RSD) on analytes’ responses: used to evaluate the repeatability and calculated on all targets for the two replicates of the average point. A %RSD value <20 is usually considered acceptable;
- % Explained variance: usually considered satisfactory if ≥80%, it expresses the fraction of the total variation in response that the model can explain. It is correlated to residuals, showing how each experimental data fits its theoretical position in the model projection [36];
- Coefficient significance: evaluated based on its value and sign, indicating a direct or inverse correlation with the chosen response. This parameter is accompanied by the confidence interval.

An in-depth discussion of the CCD model is out of the scope of this study although data is available on demand for interested readers on the Open Science Framework (OSF) website in a dedicated repository: https://osf.io/63ghp/. Table 3 lists the optimized sampling conditions for detailed VOCs profiling as resulted by the DoE application.

### 2.5. MHS-SPME Principles and Conditions

Multiple headspace extraction (MHE) is a dynamic, stepwise gas extraction approach available for the accurate quantification of volatiles from solid or heterogeneous matrices. When includes the SPME enrichment it is referred to as MHS-SPME [37,38,39,40].

For accurate quantification of analytes it can be performed as external standard calibration and requires a three-step procedure:

- exhaustive extraction of target volatiles from calibration standards or certified material covering the actual range of concentrations/amounts of real samples;
- exhaustive extraction, by successive steps, of target volatiles from samples to define HS linearity boundaries vs. HS saturation [37,40,41];
- application of the procedure to samples of interest.

A more detailed description of the MHS-SPME procedure is available in the referenced literature [27,37,40,41].

The sum of the instrumental response, here referred to as the chromatographic peak area (*A_s_*) and measured at each step of HS extraction/sampling, equals the total response (*A_T_*) as generated by the analyte amount in the sample. To estimate the cumulative instrumental response, *A_T_* Equation (1) is applied:

(1) $$A_{T}=\sum_{i=1}^{=\infty }A_{i}=A_{1}\;\frac{1}{\left(1-{\;e}^{-q}\right)}=\frac{A_{1}}{\left(1-\;\beta \right)}$$

where *A_T_* is the total response, *A_1_* is the analyte’s chromatographic area (absolute or normalized) from the first extraction/sampling step, and *q* is a constant corresponding to the response exponential decay (β) from consecutive extractions.

The *q* constant is obtained by the natural logarithm of the chromatographic peak areas vs. the number of extraction steps. By that a linear regression (Equation (2)) can be calculated:

(2) $$\ln A_{i}=a\;\left(i-1\right)+b$$

where *i* is the number of extraction/sampling steps, *b* is the intercept on the y axis, and *a* is the slope.

The β (e^–*q*^) constant is analyte-matrix dependent under specific conditions, therefore, informing the matrix effect, i.e., analyte retention into the matrix [42]. Moreover, as indicated by Kolb and Ettre [41], a β value ≤ 0.8 confirms the HS linearity assumption. When MHS-SPME is applied to calibration solutions, it provides data for external calibration. Calibration curves are then applied to estimate the accurate amount of the analyte in the sample. At this stage, by a simplified procedure, the analyte chromatographic area (absolute or normalized) after the first extraction/sampling step (*A_1_*) from the real sample is sufficient for an accurate quantification [37].

MHS-SPME was conducted on differential amounts of finely ground samples aliquots. Depending on the absolute amount of targeted aldehydes, 250, 150, 100, 50, or 25 mg ± 0.2 mg of sample powder were submitted to sampling in a 20 mL HS vial at 50 °C for 50 min. The exact amount of sample was tuned based on HS linearity achieving β values below 0.8 in all cases.

### 2.6. GC-MS System Set-Up and Analytical Conditions

The GC-MS system consisted of a MPS-2 multipurpose auto-sampler (Gerstel GmbH, Mülheim an der Ruhr, Germany) integrated with an Agilent 7890Aplus GC unit, coupled to an Agilent 5977B MS detector provided with a high efficiency ion source HES (Agilent Technologies, Little Falls, DE, USA) and operating in electron ionization mode (EI) at 70 eV. The GC transfer line was set at 270 °C and the MS scan range was 40–300 *m*/*z* with a scanning rate of 9600 amu/s.

The capillary column was a Heavy-Wax column (100% polyethylene glycol, 30 m × 0.25 mm *d_c_*, 0.25 μm *d_f_*) (Agilent Technologies). Carrier gas was helium at a constant flow of 1 mL/min. The temperature program was: from 40 °C (2 min) to 270 °C at 3.5 °C/min (5 min). SPME thermal desorption into the split/splitless GC injector port operated under the following conditions: injection mode: split, split ratio 1:5, injector temperature 270 °C, and 5 min of thermal desorption.

The *n*-alkanes calibration solution for *I^T^* determination was analyzed under the following conditions: split/splitless injector in split mode, split ratio 1:50, injection volume 1 μL.

### 2.7. Data Acquisition and Data Processing

Data were acquired by MassHunter (Agilent Technologies) and processed by Agilent MSD ChemStation version E.02.02.

Statistical analysis and chemometrics were conducted by XLSTAT 2014 (Addinsoft, New York, NY, USA) while heat-map visualization was by Gene-E (https://software.broadinstitute.org/GENE-E/ - last accessed on 10 May 2022).

## 3. Results and Discussion

This section illustrates the integrated strategy developed to obtain information about samples’ volatile fraction composition and its evolution along shelf-life with insights on the accurate amount of secondary products of FAs hydroperoxides degradation as rancidity markers. Results will be preceded by some considerations about the information potential of HS-SPME in profiling studies.

To complete the picture, the actual quantification inaccuracy of HS-SPME sampling conducted with internal standardization vs. MHS-SPME will be shown and the information capabilities of each approach commented.

### 3.1. Qualitative vs. Quantitative Profiling of Volatiles: Considerations

The food volatile fraction is a mine of functional information. Edible crops express within the volatilome [43] their distinctive phenotype, pedoclimatic impact, harvesting conditions, post-harvest treatments, shelf-life, and storage conditions [39,44,45]. Processing technologies and/or fermentation are also clearly represented through diagnostic patterns of volatiles formed within known reaction frameworks, e.g., Maillard reaction, sugars caramelization, and amino acids degradation. The detailed profiling of food volatiles becomes therefore crucial when the functional variables related-information helps in decision-making strategies and/or for new-process development. However, a crucial role is played by the analytical strategy implemented; if quantitative information is required, not all available methodologies are adequate.

Physico-chemical properties of volatiles make them suitable for gas-phase extraction approaches, i.e., headspace sampling (HS). Extraction from the vapor phase, under equilibrium or non-equilibrium conditions, provides information about components distribution and/or amount in the original sample based on compound-specific partition coefficients *K_hs_* [37], Equation (3).

(3) $$K_{hs}=\frac{C0}{Cg}$$

where: *C*_0_ is the analyte concentration in the sample and *Cg* is the analyte concentration in the vapor phase or headspace.

Within the static HS sampling procedures, HS-SPME is undoubtedly the most popular high concentration capacity (HCC) approach [46,47,48,49,50], as being easy to standardize and fully integrated with the analytical platform through automated systems. It is the ideal solution for high-throughput profiling and fingerprinting studies [44].

Since the HS-SPME system is characterized by a distribution of components across the three physical phases (i.e., the condensed phase/sample, the headspace, and the fiber polymer or composite material coating) as a function of the temperature and relative pressure; the recovery of analytes from the HS is governed by two closely related yet distinct equilibria. The condensed phase/sample vs. HS equilibrium is governed by the distribution coefficient K*_hs_*, while the fiber vs. HS equilibrium is characterized by a distribution coefficient K*_fh_*. The amount of analyte recovered by the extraction phase (*n*) at equilibrium is therefore estimated by Equation (4):

(4) $$n=\frac{K_{hs}K_{fh}V_{f}V_{s}C_{0}}{K_{hs}K_{fh}V_{f}+K_{hs}V_{h\;}+V_{s}}$$

where *C*_0_ is the analyte concentration in the sample, *K_fh_* is the fiber/HS distribution coefficient, *K_hs_* is the sample/headspace distribution coefficient, *V_s_* is the sample volume, *V_f_* is the fiber coating volume, *V_h_* is the headspace volume.

By Equation (4) it appears that the amount of an analyte extracted by the SPME is in direct proportion to its concentration in the sample, thus making HS-SPME suitable for quantitative analysis. However, the dynamics of adsorption/sorption during sampling refers to a linear relation between *n* and *C*_0_ [51]; thus quantitation is also possible in non-equilibrium conditions.

For profiling purposes, volatile components and/or markers can be cross-compared based on quantitative indicators derived by instrumental analysis; indicators can be the chromatographic peak areas (raw areas, percentage area); the peak volumes for comprehensive two-dimensional GC (GC × GC) (raw volume, percentage volume); or the normalized responses over the internal standard (IS) (normalized area, normalized volume). The latter, accepted by the scientific community for some applications [52], might be inaccurate or misleading if treated as an indicator of the analyte(s) actual amount in the sample.

Normalized responses from volatiles extracted by solid or liquid samples, do not take into consideration the matrix effect on analytes released into the HS; a characteristic that is modeled by the β constant estimated/measured with MHS-SPME in predetermined conditions. The heterogeneous composition and structure of many solid foods exert specific retention on native volatiles that can be differently partitioned (absorbed) or adsorbed into the solid particles network with consequences in their release and equilibration with the HS. In practice, due to the different physicochemical properties, volatile components may show widely different *K_hs_* values preventing the adoption of any generalized approach for their accurate quantification.

Accurate quantification of volatiles and semi-volatiles by HS can be carried out in different ways, each one including an external/internal calibration with authentic standards. External standard calibration in matrix-matched blank samples is suitable for liquid samples and has been successfully adopted for edible oils [53,54]; standard addition (SA) by spiking the sample with known incremental amounts of analyte(s) is suitable for liquid samples although in food applications it has also been proposed for solids particulate as coffee powder and dried herbs [55,56]; stable isotope dilution, a specific application of the SA (SIDA) is a common approach in *sensomics* [57]; and MHE with its flexibility has been used for both liquid and solid complex samples [12,40,58,59].

For accurate estimation of the analyte(s) amount, HS linearity conditions must be verified [37]. This condition is established when the analyte amount released by the sample/condensed phase, under the applied t/T parameters, does not saturate the HS while matching method sensitivity. In practice, within linearity conditions, the analyte concentration in the sample (*C*_0_) and its concentration in the gas phase (*C_g_*) follow a linear function. The actual range of linearity depends on *K_hs_* and the analyte activity coefficient: it generally varies between 0.1 and 1% in the sample.

Although HS linearity is easily achievable by trace and sub-trace analytes, it becomes challenging in multi-analyte quantitation. For these reasons, to enable effective multitarget profiling by HS-SPME of solid samples, an integrated strategy is mandatory. The combination of informative profiling directed to the largest number of volatiles should be accompanied by an accurate quantitative procedure that takes into account HS linearity and appropriate external calibration.

The current study combines optimized HS profiling conditions for the selected model samples, as indicated by the CCD screening, with a validated procedure for accurate quantitative assessment of secondary product of FAs hydroperoxides degradation [27].

### 3.2. Qualitative Profiling of Walnut (*Juglans regia* L. var. Chandler) Volatiles within Shelf-Life

The volatile fraction of raw walnuts accounted for about 300 detectable compounds above a response threshold of 150 counts (Total Ion Current—TIC trace). Within them, for 95 compounds it was possible to assign a putative identity based on MS spectral similarity (above 900 direct match factor—DMF value) with reference compounds collected in the NIST [60] and Wiley [61] databases and with *I^T^* coherence with tabulated values (± 10 units). Supplementary Table S1 lists targeted analytes identified in walnuts.

Chemical classes include the informative group of aldehydes and short-chain FAs; they are generally formed by cleavage of FAs hydroperoxides and are connoted by *green* and *citrus*-*like* notes for the low-molecular-weight congeners (hexanal, heptanal, and unsaturated derivatives) and *fatty* and *rancid* notes (octanal, nonanal, (*E*)-2-nonenal, butanoic acid, heptanoic acid, hexanoic acid etc.). This group was specifically monitored through quantitative MHE to follow their trend along the shelf-life of samples; results are commented on in the dedicated section.

Walnuts are also characterized by the presence of linear alcohols, some esters (2-butyl acetate, ethyl acetate, butyl benzoate, methyl hexanoate, hexyl butanoate, isobutyl isobutyrate, and butyl butanoate) and terpens/terpenoids (1,8-cineole, α-pinene, β-pinene, β-phellandrene, limonene, *m*-cymene, and *p*-cymene).

Profiling capabilities were confirmed by a comparative evaluation of existing literature data. Elmore et al. studied the volatile fraction of raw walnuts harvested in different geographical areas (China, Ukraine and Chile) while assessing the presence of 118 volatile components extracted by dynamic HS with trapping on Tenax TA cartridges [62]. Authors, by semi-quantitative assessment, found that the most abundant compounds were: hexanal followed by 1-pentanol, pentanal, and 1-hexanol. These analytes are likely formed by the oxidation of linoleic acid, which is the predominant FAs in walnuts. Of interest, the hexanal content was higher in oxidized walnuts, confirming its role as the primary marker of oxidative flavor deterioration [1,63]. Moreover, Jensen et al. positively correlated the hexanal content with *bitter* and *rancid* tastes while observing a negative correlation with *nutty* and *sweet* qualities [64]. In a recent study, Grilo and Wang [65] studied the evolution of raw walnuts along 28 weeks of storage; authors concluded that some informative volatiles have a better diagnostic role compared to other chemical indices (e.g., peroxide value PV, UV absorbance, total phenols, etc.) in discriminating walnut oxidation levels. They are pentanal, hexanal, (*E*)-2-pentenal, 3-octanone, octanal, hexanol, (*E*)-2-octenal, 1-octen-3-ol, benzaldehyde, and hexanoic acid. All these compounds were here successfully monitored by the informative HS-SPME profiling step.

Collecting information on potent odorants and key-aroma compounds adds further value to any profiling strategy; according to Liu et al. [66] who applied sensomic protocol to reveal the aroma code of raw walnuts, a total of 10 aroma compounds reported Odor Activity Values (OAVs) >1. Of them, those matching the sensory qualities of raw walnut are: (*E*)-2-nonenal (OAV = 2217) with a strong *grass*-*like* note, octanal (OAV = 769), hexanal (OAV = 753), and nonanal (OAV = 500) contributing with different extents to the *green grass* and *fruity* flavor.

**Figure 1:**
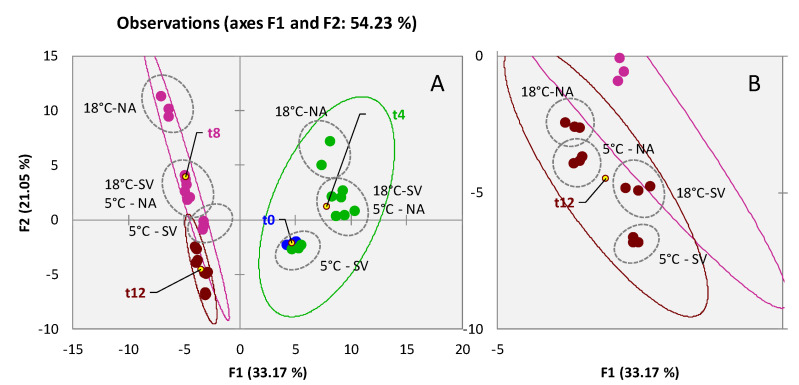
PCA scores plot based on normalized chromatographic areas from 95 targeted compounds across all analyzed walnut samples (**A**). Confidence ellipses (95% of confidence) relate to shelf-life (t0-blue; t4-green; t8-purple; t12-garnet color). In (**B**) the t12 samples are highlighted.

An unsupervised exploration of the distribution of targeted analytes provided proof that the captured volatiles’ patterns were capable of differentiating storage time and conditions in high-quality walnuts. Figure 1A shows the scores plot of a Principal Component Analysis (PCA) based on the normalized response distribution of 95 targeted compounds across all analyzed samples. The combination of PC1 and PC2 covers 54.23% of the total explained variance with a fairly clear natural clustering of samples (confidence ellipses set at 95%) according to storage time. Observing the squared cosines of the variables on F1, where samples are discriminated by storage time, besides known oxidative markers (i.e., hexanal, hexanoic acid, (*E*)-2-heptenal, 6-methyl-5-hepten-2-one, (*E*)-2-octenal, and nonanal listed in decreasing order of squared cosine value), some free FAs [octadecanoic acid, (*Z*)-octadec-9-enoic acid (oleic acid), heptadecanoic acid, tetradecanoic acid, and pentadecanoic acid listed in decreasing order of squared cosine value] suggest the triggering of lipases activity [13].

The effect of storage (i.e., temperature 5/18 °C and atmosphere by regular air NA or under vacuum SV) is mostly explained along F2 with samples subjected to less protective conditions reporting higher loadings, as detailed by samples’ tags in Figure 1A,B, the latter providing insights on the 12 months’ samples. A clearer effect of the differential impact of storage conditions on volatile markers is shown later with the quantitative profiling strategy.

As a general consideration, the walnut volatilome shows great variations (in terms of analytes relative abundance) within the first four months of storage. Up to the first time-point, the temperature and the presence of oxygen trigger several reactions; samples appear dispersed along both PCs, with apparently similar impact for the conditions 18 °C—SV and 5 °C—NA. Just after 12 months, the primary role of atmosphere composition dominates (Figure 1B) and samples stored at 18 °C under vacuum are closer to those stored at 5 °C. These results are in line with those of Cialiè Rosso [14] who studied the evolution of volatile patterns of raw hazelnuts stored in similar conditions.

### 3.3. Qualitative Profiling of Almond (*Prunus dulcis* (Mill) var. Aldrich) Volatiles within Shelf-Life

The volatile fraction of almonds accounted for about 280 detectable compounds above a response threshold of 150 counts. Within them, for 91 compounds it was possible to assign a putative identity based on MS spectral similarity (above 900 DMF value) with reference compounds collected in the NIST [60] and Wiley [61] databases and with *I^T^* coherence with tabulated values (±10 units). Supplementary Table S1 lists targeted analytes identified in almonds.

Chemical classes include the informative group of aldehydes and alcohols accounting for more than thirty different congeners. Within them, the sub-group of secondary products of lipid oxidation with a high odor impact (low OT): 1-octen-3-ol, 1-octanol, hexanal, heptanal, octanal, nonanal, decanal, (*E*)-2-heptenal and (*E*)-2-octenal are dominating.

**Figure 2:**
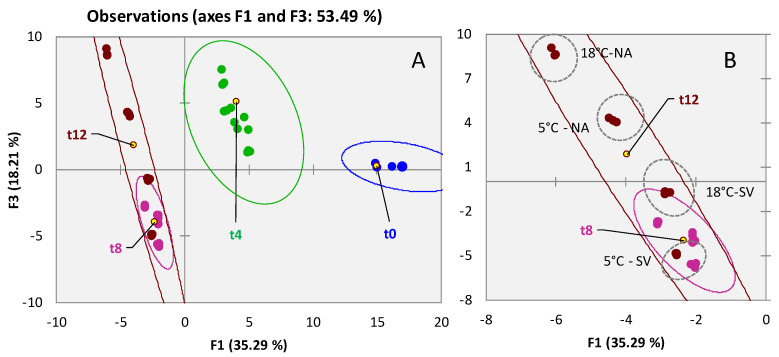
PCA scores plot based on normalized chromatographic areas from 91 targeted compounds across all analyzed almond samples (Figure 1A). Confidence ellipses (95% of confidence) relate to shelf-life (t0-blue; t4-green; t8-purple; t12-garnet color). Insight on t12 samples in (Figure 1B).

A first unsupervised exploration of the distribution of the 91 targeted analytes confirms that chemical signatures are distinctive for time and storage conditions. Figure 2A shows the PCA scores plot for the almonds sample set (*n* = 39 analyses), natural clustering of samples is driven by storage time along F1 with a negative correlation with loadings, and again along F3 where samples are distributed from low to high loadings according to storage conditions. An insight on t12 samples is provided in Figure 2B.

Observing the squared cosines of the variables on F1, where samples are discriminated by storage time, besides known oxidative markers (i.e., hexanal, octanal, (*E*)-2-octenal, nonanal, and decanal), some additional compounds deriving by kernel primary metabolites degradation were found. In particular, acetic and butyric acid are likely formed by bacterial fermentation on sugars and FAs; 2-methyl-butanal is formed by Streker degradation in leucine; medium chain aldehydes 2-ethyl-hexanal and 2-ethyl-2-hexenal are already documented in many vegetable foods as markers of viability [67,68], and (*E*)-2-hexenal and 1-hexanol are likely formed by enzymatic cleavage of FAs hydroperoxides. One key odorant has also a characteristic distribution as a function of storage time; it is benzaldehyde, released by the di-glycoside amygdalin, with a decreasing trend along shelf-life [69].

As for walnuts, also, in this case, the differential impact of storage atmosphere and temperature had an evident impact on the volatile patterns. Analytes with an informative potential along F3, and correlated to storage variables, are 1-butanol, 1-pentanol, 1-heptanol, 1-octanol, 1-octen-3-ol, heptanal, octanal, nonanal, (*E*)-2-octenal, and hexanoic acid all derived by autoxidation of fats.

Interestingly, as suggested by PCA results, it appears that storage time is connoted by a general degradation of primary metabolites that, in their turn, are forming characteristic volatile degradation products. On the other hand, within storage time points, the effect of temperature and oxygen availability (e.g., for NA conditions) has a major impact on autoxidation producing a well-known signature of FAs hydroperoxides degradation products.

Profiling results are aligned with most recent literature on raw almond volatilome [34,69,70]; with its FAs compositional profile dominated by oleic acid (62–80%), followed by linoleic acid (10–18%), palmitic (0.5–8%) and stearic (1–3%) acids, the expected pattern of volatile secondary products should include, as primary component hexanal (100%), followed by nonanal (34%), octanal (30%), and (*E*)-2-octenal (19%). This estimation was based on the data resulting from the accurate quantification of secondary products of oleic and linoleic hydroperoxides cleavage in a model system studied by Grosch, Schieberle, and co-workers, and consisting of 1 g of FA kept at 20 °C and with a FA uptake of 0.5 mole oxygen/mole [71,72].

With regard to aroma compounds, raw almonds were studied by applying the molecular sensory science protocol by Erten and Cadvallader [73]. Authors identified by Aroma Extract Dilution Analysis (AEDA) 1-octen-3-one (*mushroom* and *metallic* notes) and acetic acid (*sour*) as high-impact odorants in raw almonds accompanied by many lipid degradation derivatives including some di-unsaturated aldehydes with very low OTs [i.e., (*E*,*E*)-2,4-nonadienal and (*E*,*E*)-2,4-decadienal] hardly detectable by HS techniques. Other studies, by correlating volatile profiles with descriptive sensory analysis, highlighted the role of several additional odorants: benzaldehyde (*sweet marzipan*-*like* aroma), benzyl alcohol (*floral* and *rose*-*like* notes), 3-methyl butanal (*malty* aroma), and hexanal (*grassy* and *fatty* notes), all successfully covered by current profiling strategy.

A better understanding of the sensory impact of rancidity markers on stored almonds will arise from their quantitative determination and subsequent evaluation of the resulting OAVs (see Section 3.5.2).

### 3.4. Qualitative Profiling of Dried Pineapple (*Ananas comosus*) Volatiles within Shelf-Life

The volatile fraction of pineapple accounted for about 300 detectable compounds above a response threshold of 150 counts. Within them, for 125 analytes it was possible to assign a putative identity based on MS spectral similarity (above 900 DMF value) with reference compounds collected in the NIST [60] and Wiley [61] databases and with *I^T^* coherence with tabulated values (± 10 units). Supplementary Table S1 lists targeted analytes identified in dried pineapple.

Chemical classes include the informative group of esters accounting for more than thirty different congeners. Fresh and optimally ripened pineapples are characterized by high relative amounts of butanoic acid esters (i.e., methyl butanoate, methyl 2-methylbutanoate), ethyl hexanoate, methyl 2-methylpropanoate, and ethyl hexanoate here listed according to Montero-Calderon et al. [74] in decreasing order of relative amount. These fruity esters (*fruity*, *banana*-*like*, *pineapple* aroma qualities) are dominating the aroma blueprint of fresh pineapple, therefore, representing a key-chemical class to monitor on dried products stored up to 12 months.

Another important chemical class is that of sulfur derivatives, represented by several congeners: methanethiol (*boiled cabbage* odor), dimethyl disulphide (*alliaceous*, *cabbage*, *creamy*, *garlic* notes), dimethyl trisulfide, 3-(methylthio)-propanal/methional (*cooked potatoes* odor), methyl 3-methylthio propionate, ethyl-3-methylthio propionate and methionol (*sulfurous*, *onion like* aroma) [75].

Aldehydes are also abundant and are represented by saturated and unsaturated derivatives (hexanal, heptanal, octanal, nonanal, decanal, (*E*)-2-hexenal, (*E*)-2-heptenal, (*E*)-2-octenal) and some with aromatic rings (benzaldehyde, benzenacetaldehyde, cinnamaldehyde, 2-phenyl-2-butenal, and vanillin). Terpenes and nor-isoprenoids are represented with many compounds likely contributing to the pleasant aroma of fresh pineapple. β-ionone (*violet*-*like*) is the congener with the lowest OT; within monoterpenoids, limonene (*citrus*), 4-terpineol (*cooling*, *woody*, *earthy*), and *p*-cymene (*terpenic*, *woody*), δ-3-carene have a characteristic distribution in fresh dried fruits. To note, for pineapples several sesquiterpenes were also identified, with some of them also contributing to the time-dependent volatile signature: germacrene D; α-gurjunene; α-amorphene; α-muurolene. On the evolution of terpenes during ripening, Steingass et al. [76,77] observed a generalized decrease during maturity with a concurrent increment of some alcohols, related esters and sulfur derivatives.

**Figure 3:**
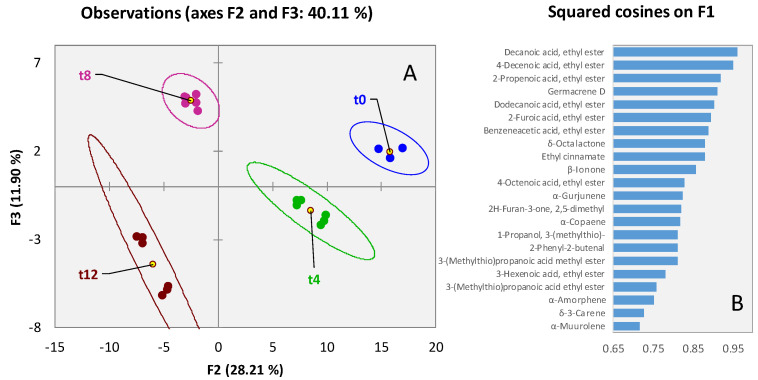
PCA scores plot based on normalized chromatographic areas from 125 targeted compounds across all analyzed dried pineapple samples (Figure 1A). Confidence ellipses (95% of confidence) relate to shelf-life (t0-blue; t4-green; t8-purple; t12-garnet color). The histogram in Figure 2B reports the squared cosines of the variables on PC1.

An unsupervised exploration of the distribution of the 125 targeted analytes confirmed the clear impact of storage time and temperature on volatile signatures. Figure 3A shows the PCA scores plot for the pineapple sample set (*n* = 21 analyses), natural clustering of samples is driven by storage time along F1 with a negative correlation with loadings. An insight into the most relevant variables contributing on PC1 is provided in Figure 3B where analytes are listed in decreasing order of squared cosines.

**Figure 4:**
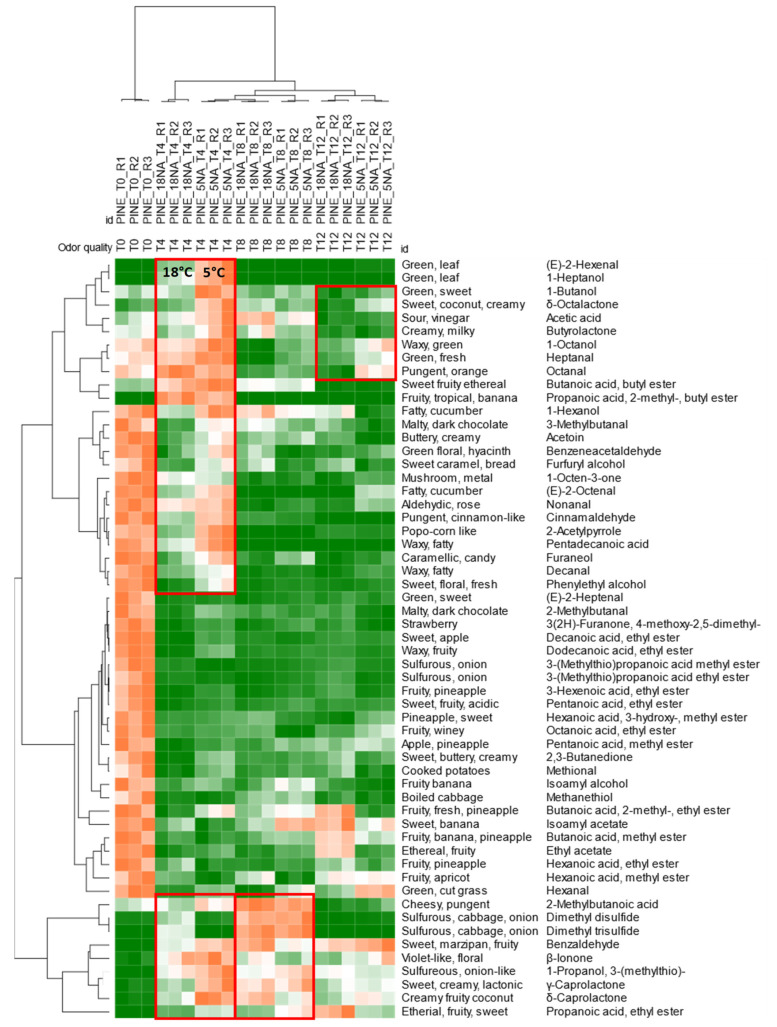
Heatmap visualization of normalized responses for potent odorants in dried pineapple samples. Hierarchical clustering is based on Pearson correlation after Z-score normalization.

The evolution of potent odorants, including those characterizing fresh pineapple aroma, along with storage time is illustrated in the heatmap of Figure 4. Hierarchical clustering is based on Pearson correlation on normalized (i.e., chromatographic areas normalized over the IS 2-methyl octynoate) peak responses after Z-score normalization. Heatmap colorization is from green (lower values) to orange (higher values).

Samples are coherently clustered according to storage time, from left to right (Figure 4) fresh dried samples at t0 form an independent group connoted by a higher relative response for most of the targeted odorants. This distribution was expected due to the nature of this pre-processed ingredient [78] that, from one side retains volatile aromatic compounds that are characteristic of the fresh fruit, but due to the lower *a_w_* loses the potential to form new aroma compounds by enzymatic activity and cell viability. The loss of potent odorants is more marked after 4 months of storage with some differences according to storage temperature. The storage at 5 °C is connoted by a higher relative distribution of some odorants (see the red squares in Figure 4) compared to the 18 °C samples. The same differential distribution can be appreciated on a sub-group of volatiles for t8 and t12 samples.

Some analytes show an opposite trend with a relative increase over the total response along shelf-life. 3-(methylthio)-1-propanol (*sulfurous*, *onion*-*like* notes), benzaldehyde (*sweet*, *marzipan*, *fruity*), β-ionone (*violet*-*like*, *floral*), γ-caprolactone (sw*eet*, *creamy*, *lactonic*), and δ-caprolactone (*creamy fruity coconut*) prevail at t8 and t12.

To better understand the contribution of fatty aldehydes to the overall perception, MHS-SPME accurate quantification was applied and OAVs were calculated (see Section 3.5.3). The next section presents the quantitative results on selected saturated and unsaturated aldehydes with low OTs.

### 3.5. Quantitative Profiling of Secondary Products of Lipid Oxidation within Shelf-Life

#### 3.5.1. Accurate Quantification of Volatile Lipid Oxidation Products in Walnuts

The accurate quantification of secondary products of lipid oxidation in walnuts was targeted to hexanal, heptanal, (*E*)-2-heptenal, octanal, (*E*)-2-octenal, nonanal, and decanal. The validated quantitative method [27], here extended to unsaturated congeners, verified the HS linearity operating on 0.250 g of ground material instead of the 1.750 g adopted for the profiling method. Under the established sampling conditions (see Section 2.5), analytes had MHE decay trends matching with recommended values (i.e., β < 0.8) and uncertainties below 20% of relative error. Table 4 reports quantitative results for walnuts; amounts expressed as ng/g correspond to the averaged value obtained from three sub-samples of the industrial batch (*n* = 3 × 2).

**Figure 5:**
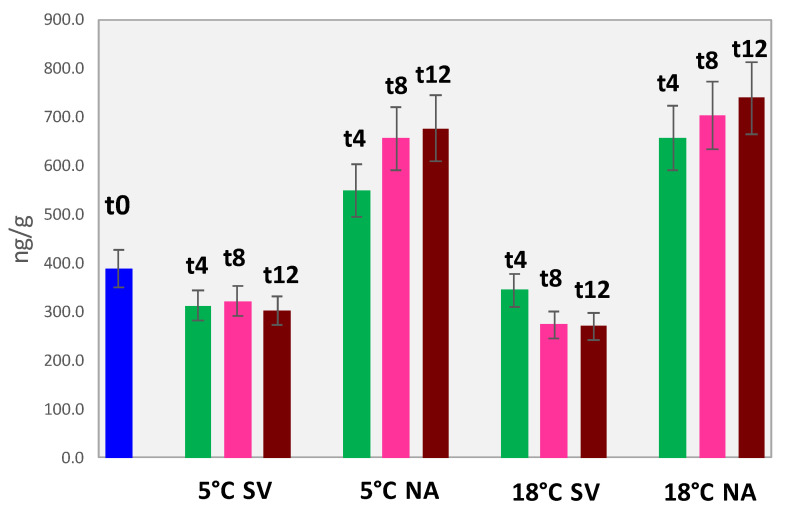
Histogram reporting hexanal equivalents (ng/g) for walnut samples analyzed at the different shelf-life time points and storage conditions.

As a general consideration, the profile of secondary products of lipid oxidation is dominated by hexanal followed by (*E*)-2-octenal, octanal, and nonanal. The impact of storage conditions along shelf life is illustrated by the histogram in Figure 5 where the hexanal equivalents were calculated by converting the amount of each target analyte to hexanal (ng/g), simplifying the evaluation of the oxidative status [27].

The OAVs for hexanal were always above the value of one (value reported in bold in Table 4) likely indicating a role of this odorant in the overall perception (hexanal OT retronasal perception in oil 75 µg/kg [79]).

The hexanal equivalents trend confirms that, at least for the autoxidation process on FAs, the storage atmosphere has a primary role; samples stored at 5 °C under vacuum have comparable amounts of oxidation products with those stored under vacuum but at 18 °C. These results, also confirmed by previous data on hazelnut storage [14], support the application of industrial strategies that limit the contact with oxygenated air while reducing the environmental impact and energy consumption of refrigeration.

From the perspective of ready-to-eat snacks combining different dried fruits and seeds, the adoption of suitable packaging combined with an inert atmosphere would be the best option.

#### 3.5.2. Accurate Quantification of Volatile Lipid Oxidation Products in Almonds

The quantification of volatile lipid oxidation markers in almonds was conducted on 0.250 g of finely ground material for the early stages of storage while it was necessary to reduce this amount to 0.050 g for the most oxidized samples (i.e., t12).

Table 5 reports quantification results for the major oxidation products (i.e., hexanal, octanal, (*E*)-2-octenal, nonanal, and decanal) accompanied by their absolute uncertainty. In almonds, according to the characteristic FAs profile, hexanal is dominating as the major product (two to three orders of magnitude higher than the others) followed by nonanal, octanal, and decanal. The OAVs were calculated and for hexanal and octanal—for a few samples—they exceeded the unity (OT retronasal perceptions of hexanal in oil was 75 µg/kg, and 50 µg/kg for octanal [79]).

**Figure 6:**
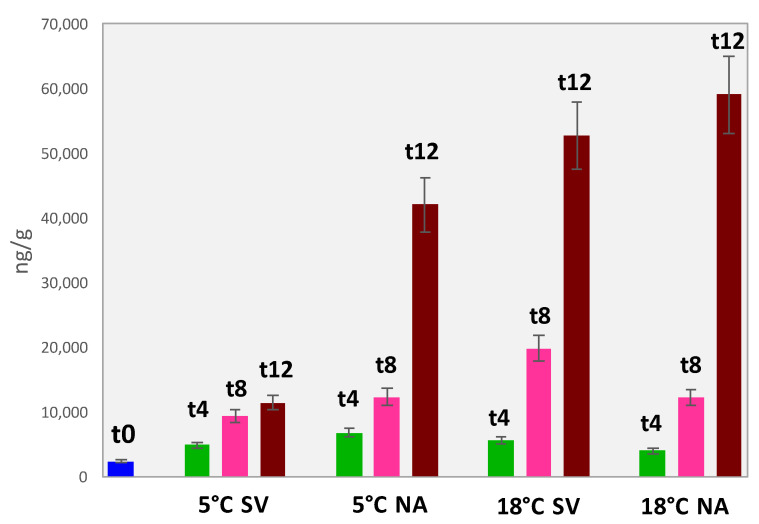
Histogram reporting hexanal equivalents (ng/g) for almond samples analyzed at the different shelf-life time points and storage conditions.

The sum of hexanal equivalents helps in delineating shelf-life trends and in evaluating the impact of storage conditions on the oxidative status. The histogram in Figure 6 well illustrates the autoxidation profile showing an exponential evolution along with shelf-life. Particularly between t8 and t12, the absolute amount of hexanal equivalent has a three to four-fold change when higher temperatures (18 °C SV and NA) or normal atmosphere (5° NA) are applied.

Interestingly, these trends were not so clearly defined in profiling data where the higher amount of sample (i.e., 1.75 g) analyzed produced a saturation of the HS—at least for the major products of oxidation.

In the case of almonds, the synergic effect of storage temperature at 5 °C and the absence of oxygen (5 °C SV), had a decisive impact on the sample’s quality, keeping the rancidity at 12 months quite low.

#### 3.5.3. Accurate Quantification of Volatile Lipid Oxidation Products in Dried Pineapples

Although dried pineapples are not characterized by a high-fat content (1–3% on dry weight), FAs profile includes oleic (25–40%), linoleic (5–30%), and linolenic (5–20%) acids with larger variations as a function of cultivar and ripening stages [80]. The auto-oxidation of this fraction, induced by the freeze-thawing process, has an impact on the overall sensorial quality as also documented by Kaewtathip and Charoenrein [81].

**Figure 7:**
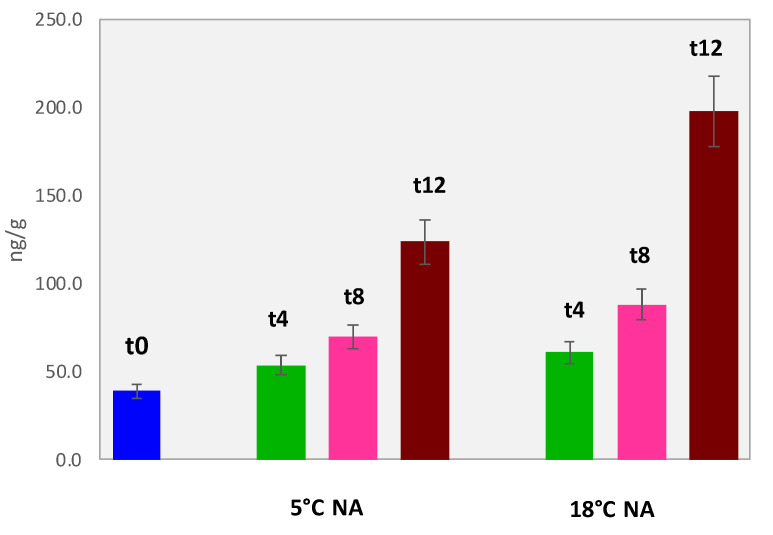
Histogram reporting hexanal equivalents (ng/g) for pineapple samples analyzed at the different shelf-life time points and storage conditions.

The quantification of lipid oxidation markers in dried pineapples was conducted on 0.250 g of finely ground material. Results are reported in Table 6 while trends as hexanal equivalents are visualized in Figure 7.

The primary product of FAs hydroperoxides degradation was nonanal followed by decanal, octanal, and hexanal. The hexanal equivalents reflect the proximate composition of this ingredient that showed the lowest amount of oxidation products compared to the others.

The trends of hexanal equivalents along shelf-life are shown by histograms in Figure 7. The amount of oxidation products at t12 is two-fold that of t0 if refrigeration is applied; at 12 months and 18 °C of storage in a normal atmosphere, the hexanal equivalent is five-fold higher.

In a ready-to-eat snack portion, as expected, quality degradation due to rancid and fatty notes would not be modulated by low-fat dried fruits, although the release of oxidation products along shelf-life is not a negligible phenomenon.

The next paragraph briefly discusses the estimation error when target analytes trends are monitored through normalized responses or inaccurate quantitative descriptors instead of absolute concentrations.

### 3.6. Quantification Error with Headspace Saturation

Regarding quantification errors that might occur quantifying analytes released by heterogeneous samples, Stilo et al. [44] have recently compared the results of an internal standardization procedure conducted by HS-SPME on extra-virgin olive oil vs. the accurate amounts obtained by MHS-SPME with external calibration. For many analytes (i.e., fifteen markers including potent odorants and geographical tracers), the % relative error (RE%) taking MHS-SPME as reference for comparison, was on average 208% achieving +538% for (*E*)-2-hexenal. This analyte is generally dominating the volatile fraction of extra-virgin olive oil, its presence responsible for the *green* and *fruity* notes [82], for its accurate quantification, the HS linearity should be carefully checked. In the cited study, to match linearity conditions, 0.100 g of oil should be sampled.

In delineating the combined profiling strategy of this study, optimal sampling conditions capable to maximize the information potential of the analysis were derived by a rational CDD approach that indicated—as expected—that higher amounts of sample matrix (1.750 g) provide good coverage of the volatilome information potential. However, such conditions might have a dramatic impact on the dynamic range of the method; for highly abundant analytes variations in the upper part of the range are not properly captured.

To provide proof of evidence of the actual error, linear regression analysis has been conducted on the response data from the profiling strategy vs. the accurate amounts derived by MHS-SPME. In particular, the normalized response for target analytes at each time point (variable *y*) has been calculated and related to the sum of hexanal equivalents in ng/g determined by MHS-SPME (variable *x*). The three matrices were treated separately due to the different sampling conditions applied for profiling (see Section 2.4).

**Figure 8:**
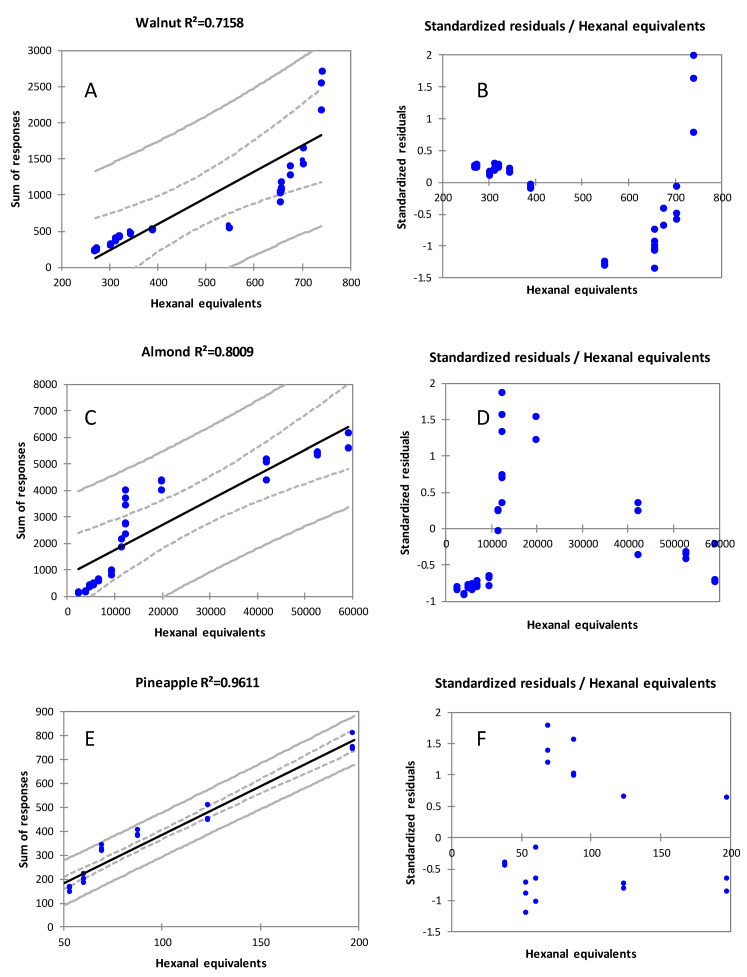
Linear regression analysis on normalized response data (variable *y*) vs. the accurate amount of rancidity markers expressed as hexanal equivalents ng/g (variable *x*). Coefficient of determination (*R^2^*) and standardized residuals are reported to complete the quality evaluation of results.

Results are visually summarized in Figure 8, where on the left are reported the regression curves (including confidence boundaries 95%) and determination coefficients (*R^2^*) (Figure 8A walnuts; Figure 8C almond; Figure 8E pineapple) while on the right side are reported the standardized residuals (Figure 8B walnuts; Figure 8D almond; Figure 8F pineapple). Except for pineapple, where the total amount of rancidity markers was very low and did not require the modulation of the sample amount to match HS linearity, for walnut and almonds, where for the correct quantification the amount of sample was varied between 0.250 and 0.050 g, there is not any linear correlation between the two variables. Moreover, as additional sources of error, it has to be considered that for target analytes both the MS response factors and *K_HS_* might be very different.

A careful exploration of the raw data indicates that within the pattern of rancidity markers, hexanal shows the largest variation due to its higher volatility and relative abundance in walnut and almond samples, thereby leveraging the normalized response data. On the other hand, minor components (heptanal, octanal, decanal and unsaturated congeners) characterized by relatively lower volatility, have a minor contribution on the cumulative response although their amounts are not negligible.

## 4. Conclusions

The study addressed the challenging scenario of informative profiling of volatiles in high-quality ingredients for confectionery and the food industry. 

To match the different investigation needs: (i) the precise capture of the volatile signature including quality markers and potent odorants responsible for the positive and negative attributes; (ii) the suitable dynamic range of the method capable to delineate analytes evolution along shelf life; (iii) the accurate amount estimation of rancidity markers; a combined strategy based on different HS sampling conditions is mandatory.

By DoE, implemented by CCD, informative profiling is possible and provides data to benchmark the quality of fresh samples and monitor their fluctuations along shelf life. This strategy implements HS-SPME with multiple ISs adopted for both analytical system performance evaluation (i.e., α/β-tujones RSD% variations) and response data normalizations (i.e., 2-methyl octynoate). By operating on a higher amount of samples (1.750 g) a wider range of analytes is captured, although for major components saturation might occur while compressing the dynamic range of responses. For accurate quantification of rancidity markers, the same analytical system is programmed to operate in MHS-SPME with a lower amount of sample (0.250–0.050g depending on the analytes’ actual concentration and the matrix effect) but providing accurate and robust data with inter-laboratory transferability and possibility for accreditation under ISO 17025 norm [83].

## Figures and Tables

**Table 1:** List of analyzed samples together with their characteristics and acronyms.

			Storage			
Sample Type	Acronym	Shelf-Life	Temperature	Atmosphere	Subsamples	N° Samples
Walnut	WAL	0 months—t0	-	-	R1, R2, R3	3
Walnut	WAL	4 months—t4	5 °C	NA, SV	R1, R2, R3	6
Walnut	WAL	4 months—t4	18 °C	NA, SV	R1, R2, R3	6
Walnut	WAL	8 months—t8	5 °C	NA, SV	R1, R2, R3	6
Walnut	WAL	8 months—t8	18 °C	NA, SV	R1, R2, R3	6
Walnut	WAL	12 months—t12	5 °C	NA, SV	R1, R2, R3	6
Walnut	WAL	12 months—t12	18 °C	NA, SV	R1, R2, R3	6
Almond	ALM	0 months—t0	-	-	R1, R2, R3	3
Almond	ALM	4 months—t4	5 °C	NA, SV	R1, R2, R3	6
Almond	ALM	4 months—t4	18 °C	NA, SV	R1, R2, R3	6
Almond	ALM	8 months—t8	5 °C	NA, SV	R1, R2, R3	6
Almond	ALM	8 months—t8	18 °C	NA, SV	R1, R2, R3	6
Almond	ALM	12 months—t12	5 °C	NA, SV	R1, R2, R3	6
Almond	ALM	12 months—t12	18 °C	NA, SV	R1, R2, R3	6
Pineapple	PINE	0 months—t0	-	-	R1, R2, R3	3
Pineapple	PINE	4 months—t4	5 °C	NA	R1, R2, R3	3
Pineapple	PINE	4 months—t4	18 °C	NA	R1, R2, R3	3
Pineapple	PINE	8 months—t8	5 °C	NA	R1, R2, R3	3
Pineapple	PINE	8 months—t8	18 °C	NA	R1, R2, R3	3
Pineapple	PINE	12 months—t12	5 °C	NA	R1, R2, R3	3
Pineapple	PINE	12 months—t12	18 °C	NA	R1, R2, R3	3

**Table 2:** Planning of experiments by CCD model.

Exp #	Amount (g)	Time (min)	Temperature (°C)
1	−1 (0.5)	−1 (30)	−1 (30)
2	−1 (0.5)	−1 (30)	+1 (60)
3	−1 (0.5)	0 (45)	0 (45)
4	−1 (0.5)	+1 (60)	−1 (30)
5	−1 (0.5)	+1 (60)	+1 (60)
6	0 (1.75)	-α (20)	0 (45)
7	0 (1.75)	0 (45)	−1 (30)
8	0 (1.75)	0 (45)	0 (45)
9	0 (1.75)	0 (45)	0 (45)
10	0 (1.75)	0 (45)	+α (70)
11	0 (1.75)	+α (70)	0 (45)
12	+ α (3.85)	0 (45)	0 (45)
13	+1 (3)	−1 (30)	−1 (30)
14	+1 (3)	−1 (30)	+1 (60)
15	+1 (3)	+1 (60)	−1 (30)
16	+1 (3)	+1 (60)	+1 (60)

**Table 3:** List of optimal sampling conditions as resulted by DoE for VOCs profiling.

Sample	Amount	Temperature	Time
Walnut	1.75 g	40 °C	60 min
Almond	1.75 g	50 °C	45 min
Pineapple	1.75 g	60 °C	45 min

**Table 4:** Amounts expressed as ng/g for quantified targeted aldehydes in walnut samples. In bold analytes whose concentration exceeds the OTs (OAV > 1).

Amount ng/g (Averaged over 3 Replicates/3 Batches ± Absolute Uncertainty)															
Sample ID	Hexanal		Heptanal		(*E*)-2-Heptenal		Octanal		(*E*)-2-Octenal		Nonanal		Decanal		Hexanal Eq.
WAL_T0	**377.8**	**±34.0**	**≤LOD-**		**3.3**	**±0.6**	1.5	±0.1	3.6	±0.4	1.2	±0.1	5.6	±0.5	389.2
														
WAL_5NA_T4	**532.5**	±47.9	≤LOD		4.4	±0.8	3.4	±0.3	4.8	±0.5	3.9	±0.4	5.3	±0.5	549.0
WAL_5NA_T8	**589.5**	±53.1	2.7	±0.3	≤LOD		25.9	±2.5	36.1	±3.5	8.2	±0.8	13.8	±1.4	655.4
WAL_5NA_T12	**607.6**	±54.7	1.0	±0.1	≤LOD		19.7	±1.9	31.1	±3.1	26.5	±2.6	13.7	±1.3	676.1
														
WAL_5SV_T4	**301.9**	±27.2	≤LOD		1.2	±0.2	1.7	±0.2	4.6	±0.5	1.0	±0.1	4.7	±0.5	311.7
WAL_5SV_T8	**277.0**	±24.9	1.6	±0.2	≤LOD		9.5	±0.9	28.6	±2.8	2.3	±0.2	18.0	±1.8	321.7
WAL_5SV_T12	**260.9**	±23.5	1.2	±0.1	≤LOD		8.0	±0.8	30.7	±3.0	3.5	±0.3	11.0	±1.1	302.1
														
WAL_18NA_T4	**565.4**	±50.9	≤LOD		21.6	±3.9	15.5	±1.5	22.8	±2.2	16.4	±1.6	47.2	±4.6	656.7
WAL_18NA_T8	**650.0**	±58.5	1.9	±0.2	≤LOD		11.4	±1.1	30.0	±2.9	8.9	±0.9	19.1	±1.9	702.9
WAL_18NA_T12	**672.5**	±60.5	3.0	±0.3	≤LOD		24.0	±2.4	32.8	±3.2	15.8	±1.5	13.3	±1.3	739.6
														
WAL_18SV_T4	**320.8**	±28.9	≤LOD		2.6	±0.5	1.0	±0.1	11.5	±1.1	10.9	±1.1	5.1	±0.5	344.0
WAL_18SV_T8	**235.8**	±21.2	2.0	±0.2	≤LOD		5.8	±0.6	27.1	±2.7	2.0	±0.2	13.4	±1.3	273.6
WAL_18SV_T12	**224.0**	±20.2	1.4	±0.1	≤LOD		4.4	±0.4	34.5	±3.4	8.0	±0.8	12.3	±1.2	269.7

**Table 5:** Amounts expressed as ng/g for quantified targeted aldehydes in almond samples. In bold are the analytes whose concentration exceeds the OTs (OAV > 1).

Amount ng/g (Averaged over 3 Replicates/3 Batches ± Absolute Uncertainty)											
Sample ID	Hexanal		Octanal		(*E*)-2-Octenal		Nonanal		Decanal		Hexanal Eq.
ALM_T0	**2345.6**	**±187.6**	**10.8**	±0.9	1.0	±0.1	10.6	±0.8	1.4	±0.1	2363.2
											
ALM_5NA_T4	**6724.8**	±538.0	13.6	±1.1	2.4	±0.2	16.0	±1.3	5.6	±0.4	6752.2
ALM_5NA_T8	**12303.6**	±984.3	21.7	±1.7	10.0	±0.8	32.6	±2.6	2.5	±0.2	12353.1
ALM_5NA_T12	**41888.8**	±3351.1	**52.9**	±4.2	43.3	±3.5	99.6	±8.0	4.2	±0.3	42037.4
											
ALM_5SV_T4	**4843.5**	±387.5	16.5	±1.3	1.9	±0.2	11.9	±1.0	5.8	±0.5	4870.0
ALM_5SV_T8	**9319.7**	±12.5	10.5	±13.9	1.9	±0.2	13.88	±1.1	1.93	±0.2	9340.5
ALM_5SV_T12	**11423.1**	±12.5	**57.7**	±13.9	17.2	±1.4	17.5	±1.4	1.8	±0.1	11454.7
											
ALM_18NA_T4	**3982.7**	±318.6	13.6	±1.1	1.4	±0.1	14.7	±1.2	6.4	±0.5	4008.9
ALM_18NA_T8	**12245.0**	±979.6	16.3	±1.3	10.0	±0.8	64.6	±5.2	3.7	±0.3	12313.5
ALM_18NA_T12	**58902.4**	±4712.2	**84.1**	±6.7	57.8	±4.6	104.3	±8.3	5.4	±0.4	59090.9
											
ALM_18SV_T4	**5655.0**	±452.4	13.6	±1.1	2.4	±0.2	12.3	±1.0	4.1	±0.3	5678.8
ALM_18SV_T8	**19775.5**	±1582.0	18.9	±1.5	14.5	±1.2	37.4	±3.0	2.7	±0.2	19829.8
ALM_18SV_T12	**52526.2**	±4202.1	**75.2**	±6.0	58.6	±4.7	100.9	±8.1	10.3	±0.8	52709.2

**Table 6:** Amounts expressed as ng/g for quantified targeted aldehydes in dried pineapple samples. In bold analytes whose concentration exceeds the OTs (OAV > 1).

Amount ng/g (Averaged over 3 Replicates/3 Batches ± Absolute Uncertainty)													
Sample ID	Hexanal		Heptanal		(*E*)-2-Heptenal		Octanal		Nonanal		Decanal		Hexanal Eq.
PINE_T0	**8.4**	**±0.8**	**1.0**	**±0.1**	≤LOD		7.5	±0.7	18.7	±1.8	16.6	±1.6	39.1
												
PINE_5NA_T4	10.2	±1.0	1.2	±0.1	≤LOD		8.7	±0.8	19.1	±1.8	34.4	±3.2	53.6
PINE_5NA_T8	15.9	±1.5	0.9	±0.1	2.4	±0.2	13.2	±1.2	19.7	±1.8	41.9	±3.9	69.8
PINE_5NA_T12	67.1	±6.3	2.8	±0.3	1.2	±0.1	14.8	±1.4	32.4	±3.0	29.1	±2.7	123.7
												
PINE_18NA_T4	12.6	±1.2	1.3	±0.1	≤LOD		10.1	±0.9	21.9	±2.0	37.3	±3.5	61.0
PINE_18NA_T8	25.3	±2.4	0.7	±0.1	2.0	±0.2	15.0	±1.4	20.6	±1.9	53.4	±5.0	88.1
PINE_18NA_T12	76.0	±7.1	7.9	±0.7	3.4	±0.3	16.5	±1.5	94.2	±8.8	50.4	±4.7	197.7

## Data Availability

Data has been uploaded to the Open Science Framework (OSF) website in a dedicated repository: https://osf.io/63ghp/. The access is made available on request.

## Supplementary Materials

The following supporting information can be downloaded at: https://www.mdpi.com/article/10.3390/foods11193111/s1, Table S1: List of VOCs putatively identified in analyzed samples together with the CAS Registry Number, experimental IT determined on a polar stationary phase (i.e., Heavy-Wax from Agilent Technologies—equivalent to Carbowax 20 M), odor quality and presence in the specified sample.
