# Supplementary material for: Integrated Strategy for Informative Profiling and Accurate Quantification of Key-Volatiles in Dried Fruits and Nuts: An Industrial Quality Control Perspective
Source: Foods. 2022 Oct 6;11(19):3111. doi: 10.3390/foods11193111 (PMC9563258; doi:10.3390/foods11193111)
Supplement: Supplementary file 1 [file foods-11-03111-s001.zip › foods-1940283-supplementary.pdf]

foods

MDPI

*Supplementary material*

# Integrated Strategy for Informative Profiling and Accurate Quantification of Key-Volatiles in Dried Fruits and Nuts: An Industrial Quality Control Perspective

**Andrea Caratti, Simone Squara, Federico Stilo, Sonia Battaglino, Erica Liberto, Irene Cincera, Giuseppe Genova, Nicola Spigolon, Carlo Bicchi and Chiara Cordero**

**Supplementary Table S1**: List of VOCs putatively identified in analyzed samples together with the CAS Registry Number, experimental $I^T$ determined on a polar stationary phase (i.e., Heavy-Wax from Agilent Technologies – equivalent to Carbowax 20M), odor quality and presence in the specified sample.

| Compound Name | CAS | $I^T$ | Odor Quality | Walnut | Almond | Pineapple |
|---|---|---|---|---|---|---|
| Pentane | 109-66-0 | 500 | | x | x | |
| Hexane | 110-54-3 | 600 | | x | x | |
| Methanethiol | 74-93-1 | 688 | Boiled cabbage | | | x |
| Heptane | 142-82-5 | 700 | | x | | |
| 2-Methyl-pentane | 107-83-5 | 754 | | x | x | |
| Octane | 111-65-9 | 800 | | | | x |
| Acetone | 67-64-1 | 814 | | | | x |
| 2-Octene | 111-67-1 | 854 | | x | x | |
| Butanal | 123-72-8 | 877 | Chocolate, cocoa | x | x | |
| Ethyl acetate | 141-78-6 | 899 | Ethereal, fruity | x | x | x |
| 2-Butanone | 78-93-3 | 903 | Ethereal, fruity | x | x | x |
| 2-Methyl-butanal | 96-17-3 | 915 | Malty, dark chocolate | x | x | x |
| Isopropyl alcohol | 67-63-0 | 920 | | x | x | |
| 3-Methyl-butanal | 590-86-3 | 927 | Malty, dark chocolate | x | x | x |
| Ethanol | 64-17-5 | 937 | | x | x | x |
| Propanoic acid, ethyl ester | 105-37-3 | 951 | Etherial, fruity, sweet | | | x |
| 2-Ethyl-furan | 3208-16-0 | 955 | Chemical, beany | x | | |
| Pentanal | 110-62-3 | 973 | Fermented, winey | x | x | x |
| Butyl ether | 142-96-1 | 974 | | x | | x |
| Dibutyl ether | 142-96-1 | 974 | | | x | |
| 2,3-Butanedione | 431-03-8 | 980 | Sweet, buttery, creamy | | | x |
| 2-Propenoic acid, ethyl ester | 140-88-5 | 980 | | | | x |
| 2-Pentanone | 107-87-9 | 983 | Fruity, banana | x | | |
| Acetic acid, sec-butyl ester | 105-46-4 | 988 | | x | | |
| Butanoic acid, methyl ester | 623-42-7 | 992 | Fruity, banana, pineapple | | | x |
| Decane | 124-18-5 | 1000 | | x | x | x |
| α-Pinene | 80-56-8 | 1024 | Herbal, woody | x | x | |
| β-Ocimene | 13877-91-3 | 1031 | Floral, green | | | x |
| Toluene | 108-88-3 | 1034 | | x | x | x |
| Isovaleric acid, ethyl ester | 108-64-5 | 1064 | | | | x |
| Dimethyl disulfide | 624-92-0 | 1065 | Sulfurous, cabbage, onion | | | x |
| Camphene | 79-92-5 | 1068 | | | x | |

| | | | | | | |
|---|---|---|---|---|---|---|
| Butanoic acid, 2-methyl-, ethyl ester | 7452-79-1 | 1073 | Fruity, fresh, pineapple | | | x |
| Hexanal | 66-25-1 | 1080 | Green, cut grass | x | x | x |
| Pentanoic acid, methyl ester | 624-24-8 | 1081 | Apple, pineapple | | | x |
| Isobutyl alcohol | 78-83-1 | 1086 | | x | | |
| Isobutyl isobutyrate | 97-85-8 | 1094 | | x | x | |
| 2-Methyl-2-butenal | 1115-11-3 | 1097 | Cocoa, phenolic | x | | |
| (*E*)-2-Pentenal | 1576-86-9 | 1103 | Green fruity apple | x | | |
| β-Pinene | 127-91-3 | 1113 | Herbal, pine | x | x | |
| Isoamyl acetate | 123-92-2 | 1115 | Sweet, banana | | | x |
| Ethylbenzene | 100-41-4 | 1116 | Phenol, spice | x | | |
| p-Xylene | 106-42-3 | 1129 | | x | x | x |
| 1-Butanol | 71-36-3 | 1134 | Fruity, balsamic | x | x | x |
| Pentanoic acid, ethyl ester | 539-82-2 | 1134 | Sweet, fruity, acidic | | | x |
| 1-Methoxy-2-propanol | 107-98-2 | 1135 | | | x | |
| 2-Butylfuran | 4466-24-4 | 1138 | | x | | |
| δ-3-Carene | 13466-78-9 | 1144 | Citrus, terpenic | x | x | x |
| Propanoic acid, 2-methyl-, butyl ester | 97-87-0 | 1154 | Fruity, tropical, banana | | | x |
| β-Myrcene | 123-35-3 | 1161 | Spicy, peppery | | x | x |
| 2-Methyl-2-pentenal | 623-36-9 | 1177 | Green, pungent | x | | |
| Hexanoic acid, methyl ester | 106-70-7 | 1184 | Fruity, apricot | | | x |
| Heptanal | 111-71-7 | 1185 | Green, fresh | x | x | x |
| o-Xylene | 95-47-6 | 1186 | Geranium | x | x | |
| Dodecane | 112-40-3 | 1200 | | x | | x |
| Limonene | 138-86-3 | 1205 | Citrus | x | x | x |
| 1,8-Cineole | 470-82-6 | 1212 | | x | x | |
| β-Phellandrene | 555-10-2 | 1215 | | x | x | |
| 2-Ethyl-2-hexenal | 123-05-7 | 1216 | | x | x | x |
| 2-Ethyl-hexanal | 123-05-7 | 1216 | | x | x | |
| Isoamyl alcohol | 123-51-3 | 1217 | Fruity banana | x | x | x |
| (*E*)-2-Hexenal | 6728-26-3 | 1220 | Green, leaf | | x | x |
| Butanoic acid, butyl ester | 109-21-7 | 1221 | Sweet fruity ethereal | x | x | x |
| 2-Hexenal | 505-57-7 | 1225 | | x | | |
| 2-Pentyl-furan | 3777-69-3 | 1230 | Fruity, vegetable | x | x | |
| Hexanoic acid, ethyl ester | 123-66-0 | 1232 | Fruity, pineapple | | | x |
| 1-Pentanol | 71-41-0 | 1252 | Fermented, fusel | x | x | |
| m-Cymene | 535-77-3 | 1267 | | | x | |
| p-Cymene | 99-87-6 | 1272 | Terpenic, wood | x | x | x |
| Isoamyl-2-methyl butyrate | 27625-35-0 | 1274 | | | | x |
| Acetoin | 513-86-0 | 1280 | Buttery, creamy | | | x |
| 2-Octanone | 111-13-7 | 1287 | Dairy, waxy | x | | |
| α-Terpinolene | 586-62-9 | 1288 | | | | x |
| Octanal | 124-13-0 | 1289 | Pungent, orange | x | x | x |
| 3-Hexenoic acid, ethyl ester | 2396-83-0 | 1290 | Fruity, pineapple | | | x |
| 1-Octen-3-one | 4312-99-6 | 1305 | Mushroom, metal | | x | x |
| (E)-2-Heptenal | 18829-55-5 | 1334 | Green, sweet | x | x | x |
| 6-Methyl-5-hepten-2-one | 110-93-0 | 1337 | Citrus, lemongrass | x | x | x |

| | | | | | | |
|---|---|---|---|---|---|---|
| 4-Hydroxy-4-methyl-2-pentanone | 123-42-2 | 1352 | | | | x |
| 1-Hexanol | 111-27-3 | 1363 | Herbal, ethereal | x | x | x |
| Dimethyl trisulfide | 3658-80-8 | 1373 | | | | x |
| Nonanal | 124-19-6 | 1398 | Aldehydic, rose | x | x | x |
| 2-Butoxy ethanol | 111-76-2 | 1405 | | x | x | x |
| Butanoic acid, hexyl ester | 2639-63-6 | 1406 | | x | x | |
| Amilfenol | 80-46-6 | 1417 | | | | x |
| (E)-2-Octenal | 2548-87-0 | 1434 | Fatty, cucumber | x | x | x |
| Octanoic acid, ethyl ester | 106-32-1 | 1440 | Fruity, winey | | | x |
| Acetic acid | 64-19-7 | 1449 | Sour, vinegar | x | x | x |
| 1-Octen-3-ol | 3391-86-4 | 1451 | Earthy, mushroom | x | x | |
| 1-Heptanol | 111-70-6 | 1465 | Green, leafy | x | x | x |
| Methional | 3268-49-3 | 1466 | Cooked potatoes | | | x |
| 4-Octenoic acid, ethyl ester | 138234-61-4 | 1470 | | | | x |
| 2H-Furan-3-one, 2,5-dimethyl | 14400-67-0 | 1483 | | | | x |
| 2-Ethyl-1-hexanol | 104-76-7 | 1484 | Citrus, fresh | x | x | |
| $\alpha$-Copaene | 3856-25-5 | 1488 | Spicy, woody | | | x |
| $\alpha$-Muurolene | 10208-80-7 | 1497 | Woody | | | x |
| Decanal | 112-31-2 | 1498 | Waxy, fatty, sweet | x | x | x |
| 2-Acetylfuran | 1192-62-7 | 1501 | | | | x |
| 3-(Methylthio)propanoic acid methyl ester | 13532-18-8 | 1510 | Sulfurous, onion | | | x |
| Benzaldehyde | 100-52-7 | 1520 | Sweet, marzipan, fruity | x | x | x |
| $\alpha$-Gurjunene | 489-40-7 | 1520 | | | | x |
| Propanoic acid | 79-09-4 | 1534 | | | | x |
| (E)-2-Nonenal | 18829-56-6 | 1540 | Green grass | x | x | x |
| 1-Octanol | 111-87-5 | 1556 | Waxy, green | x | x | x |
| 5-Methyl-2-furfural | 620-02-0 | 1570 | | | | x |
| 3-(Methylthio)propanoic acid ethyl ester | 13327-56-5 | 1571 | Sulfurous, onion | | | x |
| 3(2H)-Furanone, 4-methoxy-2,5-dimethyl- | 4077-47-8 | 1584 | Strawberry | | | x |
| 4-Terpineol | 562-74-3 | 1598 | Cooling, woody, earthy | | | x |
| 2-Furoic acid, ethyl ester | 614-99-3 | 1611 | | | | x |
| 4-Decenoic acid, methyl ester | 1191-02-2 | 1611 | | | | x |
| Butyrolactone | 96-48-0 | 1617 | Creamy, milky | | | x |
| 2-(2-Ethoxyethoxy)-ethanol | 111-90-0 | 1619 | | x | x | |
| Hexanoic acid, 3-hydroxy-, methyl ester | 21188-58-9 | 1631 | Pineapple, sweet | | | x |
| p-Menthan-3-ol | 1490-04-6 | 1631 | | x | | |
| Butyric acid | 107-92-6 | 1637 | | x | x | |
| Decanoic acid, ethyl ester | 110-38-3 | 1638 | Sweet, apple | | | x |
| Benzeneacetaldehyde | 122-78-1 | 1648 | Green floral, hyacinth | | | x |
| 2-Methylbutanoic acid | 116-53-0 | 1657 | Cheesy, pungent | | | x |
| 4-Decenoic acid, ethyl ester | 7367-84-2 | 1659 | | | | x |
| Furfuryl alcohol | 98-00-0 | 1669 | Sweet caramel, bread | x | | x |
| $\alpha$-Amorphene | 20085-19-2 | 1692 | | | | x |
| $\gamma$-Caprolactone | 695-06-7 | 1703 | Sweet, creamy, lactonic | | | x |

| | | | | | | |
|---|---|---|---|---|---|---|
| Germacrene D | 23986-74-5 | 1705 | Woody, spice | | | x |
| 1-Propanol, 3-(methylthio)- | 505-10-2 | 1720 | SuIfureous, onion-like | | | x |
| β-Selinene | 17066-67-0 | 1729 | | | | x |
| Nicotinic acid, methyl ester | 93-60-7 | 1743 | | | | x |
| Naphthalene | 91-20-3 | 1749 | | x | x | |
| 3-methyl-2(5H)-furanone | 22122-36-7 | 1750 | | | x | |
| Methyl 5-acetoxyhexanoate | 35234-22-1 | 1759 | | | | x |
| Benzeneacetic acid, ethyl ester | 101-97-3 | 1779 | | | | x |
| δ-Valerolactone | 542-28-9 | 1780 | | x | | |
| δ-Pentalactone | 542-28-9 | 1784 | | | x | |
| 2-(2-Butoxyethoxy)-ethanol | 112-34-5 | 1796 | | x | x | |
| δ-Caprolactone | 823-22-3 | 1818 | Creamy fruity coconut | | | x |
| Dodecanoic acid, ethyl ester | 106-33-2 | 1825 | Waxy, fruity | | | x |
| Hexanoic acid | 142-62-1 | 1839 | | x | x | x |
| Benzoci acid, butyl ester | 136-60-7 | 1847 | | x | x | |
| Benzyl alcohol | 100-51-6 | 1877 | Floral, rose | | x | x |
| 2-Phenyl-2-butenal | 4411-89-6 | 1911 | | | | x |
| Phenylethyl alcohol | 60-12-8 | 1920 | Sweet, floral, fresh | | | x |
| β-Ionone | 79-77-6 | 1947 | Violet-like, floral | | | x |
| δ-Octalactone | 698-76-0 | 1965 | Sweet, coconut, creamy | | | x |
| 2-Acetylpyrrole | 1072-83-9 | 1971 | Pop-corn like | | | x |
| Cinnamaldehyde | 104-55-2 | 2007 | Pungent, cinnamon-like | x | x | x |
| Phenol | 108-95-2 | 2008 | Phenolic, plastic rubber | x | x | x |
| 1H-Pyrrole-2-carboxaldehyde | 1003-29-8 | 2032 | | | | x |
| Furaneol | 3658-77-3 | 2037 | Caramellic, candy | | | x |
| Octanoic acid | 124-07-2 | 2046 | | x | x | x |
| p-Cresol | 106-44-5 | 2089 | | | | x |
| 4-Phenyl-3-buten-2-one | 122-57-6 | 2103 | | | x | x |
| Ethyl cinnamate | 103-36-6 | 2108 | | | | x |
| 2-Phenoxyethanol | 122-99-6 | 2126 | | x | x | x |
| Eugenol | 97-53-0 | 2167 | Clove | | | x |
| Nonanoic acid | 112-05-0 | 2173 | | x | x | x |
| Methyl dihydrojasmonate | 24851-98-7 | 2237 | | x | x | x |
| 4H-Pyran-4-one, 2,3-dihydro-3,5-dihydroxy-6-methyl- | 28564-83-2 | 2274 | | | | x |
| Decanoic acid | 334-48-5 | 2281 | | x | x | |
| p-Allylphenol | 501-92-8 | 2329 | | | | x |
| Isophthalaldehyde | 626-19-7 | 2341 | | | | x |
| Phthalide | 87-41-2 | 2356 | | | x | x |
| Furfural | 84-66-2 | 2372 | Bready, brown | x | x | |
| 2H-Pyran-2,6(3H)-dione | 5926-95-4 | 2427 | | | | x |
| Benzophenone | 119-61-9 | 2462 | | | | x |
| Dodecanoic acid | 143-07-7 | 2474 | | | x | |
| Vanillin | 121-33-5 | 2566 | Vanilla.like, sweet | | | x |
| Tetradecanoic acid | 544-63-8 | 2724 | | x | x | x |
| Pentadecanoic acid | 1002-84-2 | 2822 | Waxy, fatty | x | x | x |
| Squalene | 111-02-4 | 2865 | | x | x | |
| Hexadecanoic acid | 57-10-3 | 2923 | | | x | x |

| | | | | | |
|---|---|---|---|---|---|
| Heptadecanoic acid | 506-12-7 | 3027 | x | x | |
| Octadecanoic acid | 57-11-4 | 3120 | x | x | x |
| Oleic acid | 112-80-1 | 3173 | x | x | x |
